# Open science practices among early-career human-computer interaction researchers in the US

**DOI:** 10.1371/journal.pone.0334692

**Published:** 2025-11-12

**Authors:** Tatiana Chakravorti, Sanjana Gautam, Sarah Rajtmajer

**Affiliations:** 1 Information Science and Technology, Pennsylvania State University, State College, Pennsylvania, United States of America; 2 School of Information, University of Texas at Austin, Austin, Texas, United States of America; National Taiwan University, TAIWAN

## Abstract

Many fields of science have heightened introspection in the wake of concerns around reproducibility and replicability of published findings. In recent years, the Human-Computer Interaction (HCI) community too has worked to implement policy changes and mainstream open science practices. Our work investigates early-career HCI researchers’ perceptions of open science and engagement with best practices through 18 semi-structured interviews. In particular, we study researchers with mixed methods or qualitative research backgrounds. Our findings highlight opportunities and challenges for the adoption of open science practices within HCI. Participants describe barriers such as a lack of incentives, cultural resistance, and concerns about intellectual property. However, they also identify positive trends, such as increased awareness of open science practices, evolving norms around peer review, and perceived benefits such as enhanced visibility, transparency, diversity, accessibility, collaboration, and research credibility. We offer recommendations to address these barriers and to promote transparency and openness in HCI. We suggest that relatively small changes at major conferences like the Conference on Human Factors in Computing Systems (CHI) and Computer-Supported Cooperative Work (CSCW) could meaningfully impact community norms. While our findings provide valuable insights about the open science practices of early-career HCI researchers, their applicability is limited to the USA only. In addition, interviews rely on self-reported data and are therefore subject to, e.g., recall bias. Future studies should include HCI researchers with different levels of experience and from various countries.

## Introduction

Over the past decade, concerns about transparency and rigor in Human-Computer Interaction (HCI) have grown considerably. Scholars have pointed to a range of issues, including inconsistent reporting of both quantitative and qualitative methodologies [1–4], questionable statistical practices [5,6], and broader concerns about insufficient methodological rigor [7]. Data sharing remains particularly limited [8]. For instance, a survey of CHI authors from 2018–2019 found that data sharing was exceedingly rare [9], and although some progress has been made for example an increase in interview protocol sharing from 2% in 2017 to 25% in 2022 the overall rates of raw (7%) and processed (17%) data sharing remain low at CHI [10].

These issues reflect broader concerns in the social and behavioral sciences, where large-scale replication failures have triggered a movement toward open science. This movement is for practices that emphasize transparency, reproducibility, data sharing, and inclusivity [11–14]. Philosophical and normative justifications for open science trace back to Mertonian ideals of communalism and organized skepticism [15,16], but today the movement is also shaped by pragmatic concerns around the credibility and reliability of published findings [17–22].

In response, open science communities have developed interventions to promote transparency across the research lifecycle from preregistration and open data to replication incentives and badging systems [23–25]. While ethical research practices have long been a foundational requirement across disciplines, open science extends these commitments by actively promoting greater and accessibility and transparency around how research is conducted, shared, and evaluated. Open science practices aim to reduce structural barriers, broaden participation, and amplify underrepresented voices in the scientific community [26,27]. Within the HCI community, initiatives like RepliCHI [28,29], open calls for research ethics at CSCW [30,31], and the Association for Computing Machinery – Special Interest Group on Computer–Human Interaction (ACM SIGCHI)’s encouragement of supplementary materials [32] reflect growing engagement with these concerns. However, the adoption of open science in HCI remains uneven, especially given its interdisciplinary nature and diversity of methodological traditions. Indeed, recent research has begun to highlight tensions between open science norms and qualitative research values [33], as well as psychological and structural barriers to practices like preregistration [34]. Prior work has emphasized some of these structural challenges, e.g., additional time and labor required to prepare datasets for sharing or anonymizing sensitive materials in qualitative research [35–37].

The adoption of open science in HCI is shaped by several field-specific characteristics. First, HCI relies heavily on a conference-centric publishing model, particularly at premiere venues like CHI and CSCW. Open science policies typically differ between conferences and journals; the latter are more likely to recognize, incentivize or even require, e.g., data sharing and preregistration. Second, the field’s emphasis on artifact-driven research, such as interactive systems or design prototypes, presents unique challenges around intellectual property, collaborative authorship, and the reproducibility of design processes [38,39]. Finally, HCI’s interdisciplinary and methodological diversity complicates the universal application of open science norms, which are grounded in assumptions from the natural sciences.

Despite these ongoing conversations, relatively little is known about how early-career HCI researchers, those still forming their professional norms and practices, engage with open science. Are they being trained and incentivized to adopt transparency-enhancing practices? Do they perceive open science as beneficial or burdensome? And how do these experiences differ across qualitative and quantitative researchers? This study aims to fill this gap by exploring early-career HCI researchers’ perceptions of open science. Through qualitative inquiry, we examine their experiences, motivations, and concerns, with attention to disciplinary tensions within the HCI research domain. Our work is guided by the following research questions:

- **RQ1**: What are early-career HCI researchers’ perceptions and experiences around open science practices? How do these experiences differ across qualitative vs. quantitative researchers?
- **RQ2**: What are the potential benefits and barriers to adopting open science practices according to HCI researchers?

By focusing on the perspectives of early-career scholars, this study contributes to current debates on research culture change, institutional incentives, and the future of openness.

## Related work

### Open science practices in HCI

Open science refers to a set of principles and practices aimed at increasing the transparency, accessibility, and reproducibility of research. It promotes collaboration, inclusion, and the sharing of research processes and outputs across all disciplines [40]. In the context of HCI, the adoption of open science has been uneven and is shaped by the field’s interdisciplinary nature, blending qualitative, quantitative, and design-based approaches.

#### Preregistration and study design.

Preregistration involves documenting a research plan before data collection and analysis, with the goal of clearly distinguishing confirmatory from exploratory research [41]. Preregistration helps to prevent p-hacking, selective reporting, and hindsight bias, thereby enhancing the credibility of findings [42,43]. However, in HCI, preregistration has not yet gained widespread adoption. Challenges stem from the exploratory and iterative nature of many qualitative and design-based studies, which resist rigid upfront planning. While initiatives such as Registered Reports [44] and OSF Registries are gaining traction in psychology and adjacent fields, HCI researchers have expressed concerns about the applicability of preregistration to their workflows [33,34]. However, preregistering qualitative studies is practically useful for combating dissemination bias and may incentivize researchers to consistently document the development of their studies [45].

#### Ethical recruitment and material sharing.

Ethical research practices, especially when working with marginalized communities or online trace data, are deeply entangled with transparency and accountability. Recent work has emphasized that ethical frameworks must be adapted to the context, needs, and histories of specific participant groups [46–49]. Open science practices such as protocol sharing and documentation of consent procedures can help improve ethical standards, yet they also raise concerns about participant anonymity, data sensitivity, and power imbalances. Studies in HCI have explored complex consent dynamics, including sharing data with industry partners [50], conducting research in online spaces [51], and managing ongoing consent in sensitive contexts such as sexual health studies [52]. One of the most tangible pillars of open science is the sharing of research artifacts—datasets, interview protocols, code, and materials—to enable replication and secondary analysis. Despite increased advocacy, actual sharing rates in HCI remain low. For instance, Salehzadeh et al. (2023) found that only 25% of CHI papers in 2022 shared their interview protocols, with raw and processed data sharing rates at 7% and 17% respectively. Furthermore, only 57% of papers adhered to basic consent documentation practices. Researchers often cite concerns about participant privacy, labor costs of curation, and the lack of clear incentives as barriers to broader adoption [10,53,54].

#### Peer review and incentives.

Transparent peer review has been proposed as a way to increase accountability and trust in the research process. In HCI, discussions around open peer review have emerged through community initiatives and workshops, but implementation remains limited. Some ACM venues now offer artifact evaluation and badging to encourage openness, but these programs face criticism for unclear standards and inconsistent impact [32,55]. Broader engagement with open evaluation such as disclosing reviewer identities or publishing review histories remains underdeveloped in the HCI community. Finally, scholars have highlighted the need for institutional support and incentive structures to encourage open science practices. Studies across disciplines show that open sharing of data and code correlates with increased citations and visibility [56]. In HCI, however, open practices are often undervalued in hiring, promotion, and publication processes. Efforts to integrate open science into academic training and reward systems are still emerging. Some recent work has explored gamified systems, funding policies, and community-led movements as possible levers for change [14,57,58].

### Transparency in HCI research traditions

Transparency is foundational to the credibility and reproducibility of scientific research [23,59], yet its interpretation and implementation vary significantly across research paradigms. In HCI, which encompasses both quantitative and qualitative traditions, the norms and challenges of transparency differ and often require tailored approaches. In quantitative HCI research, transparency typically emphasizes the disclosure of experimental protocols, datasets, statistical methods, and code. This enables replication and supports claims of generalizability. However, studies have shown that such transparency remains limited. For example, Wacharamanotham et al. [9] found that key elements like sample size justifications, data availability, and analysis pipelines are often omitted in published HCI papers. In contrast, transparency in qualitative HCI research centers more on methodological openness than on raw data sharing, given the sensitive and contextual nature of the data. Researchers emphasize offering enough methodological detail to support trust and credibility without compromising participant confidentiality or narrative nuance [60]. Transparency here includes practices like sharing interview protocols, reflexive documentation, and thick descriptions of analysis processes. Despite these differing emphases, both traditions share ongoing concerns about insufficient reporting standards. Prior work has identified gaps in how methods are documented, how ethical decisions are communicated, and how findings are contextualized [5,61]. Replication studies essential for testing robustness and generalizability remain rare in HCI, further challenging efforts to build cumulative knowledge [3]. While several community efforts have promoted transparency through reporting checklists, badging schemes, and replicability initiatives, there remains no comprehensive synthesis of how these practices are currently understood and enacted across HCI subfields. Our study addresses this gap by exploring how early-career HCI researchers conceptualize and navigate transparency in their work.

### Challenges to open science in qualitative research

There are important differences in the challenges of practicing open science within qualitative versus quantitative research [62]. Implementing open science policies often requires distinct approaches depending on whether the study involves qualitative or quantitative methods. While qualitative research is often positioned in contrast to quantitative methods—focusing on words rather than numbers—this distinction is overly simplistic and breaks down in practice [63]. Qualitative approaches are highly diverse, varying in data types, analytic methods, and underlying epistemological assumptions. Some methods are so distinct they defy categorization as strictly qualitative or quantitative. Current movements toward standardization, such as journal reporting guidelines, may privilege mainstream qualitative approaches and marginalize non-traditional ones. This can discourage researchers from using innovative or less conventional methods, leading to a narrowing of qualitative research practices. Despite broad acknowledgment that the qualitative–quantitative divide is outdated, it continues to shape how open science (OS) is discussed and implemented in the social and behavioral sciences. Open science policies often fail to fully consider the complexities of qualitative research, raising concerns that quantitative research will benefit at the expense of other epistemologies [64]. In other words, while open science can enhance transparency and trust, it may inadvertently favor quantitative and standard approaches, raising concerns about equity in research practices [65,66]. To make open science work well for everyone, it needs to be flexible and respectful of different ways of doing research [67].

## Methodology

We conducted 18 semi-structured interviews [68,69] with early-career HCI researchers to understand their perceptions, experiences adopting open science in their research, and challenges of these practices.

### Participant recruitment

We utilized direct emails and social media platforms such as LinkedIn and Twitter to share our recruitment message and reach a diverse population of respondents. Our target population was early-career HCI researchers such as PhD students, post-doctoral researchers, assistant professors, and industry researchers from the US. We recruited 18 participants (P1-P18), including 11 females and 7 males; 1 postdoc, 1 assistant professor, 2 industry researchers, and 14 PhD students. Participants range in age from 25-35 years. In total, 16 participants come from academia, from both R1 and R2 universities (10 participants from R1, 6 from R2). All participants work in research-intensive labs. Due to IRB policies and for the protection of participants’ privacy, we can not disclose the names of the universities or companies of our participants. Other participant details, however, are provided in Table 1. Our recruitment email and study advertisement contained information about the goals of the study, expected interview length, compensation, inclusion criteria, and a link to the screener survey (Google form). This screener survey helped us to select a diverse research group, from different professional backgrounds, different universities, different HCI research backgrounds, and genders. Three different time slots were provided by the participants for the semi-structured interview; from those, we selected one.

**Table 1:** Gender, profession, rank, and research field of interview participants.

ID	Gender	Institute	Profession	Research field
P1	Female	Academia	1st year PhD	Qualitative Method
P2	Female	Academia	2nd year PhD	Qualitative Method
P3	Male	Industry	UX Researcher	Mixed Method
P4	Female	Academia	2nd year PhD	Qualitative Method
P5	Male	Academia	3rd year PhD	Mixed Method
P6	Male	Academia	2nd year PhD	Mixed Method
P7	Female	Academia	1th year PhD	Mixed Method
P8	Female	Academia	Post Doctoral Researcher	Mixed Method
P9	Male	Academia	1th year PhD	Mixed Method
P10	Female	Academia	3rd year PhD	Qualitative Method
P11	Female	Academia	2nd year PhD	Mixed Method
P12	Male	Academia	1st year PhD	Mixed Method
P13	Female	Academia	2nd year PhD	Mixed Method
P14	Male	Academia	3rd year PhD	Qualitative Method
P15	Female	Industry	UX Researcher	Qualitative Method
P16	Female	Academia	Assistant Professor	Mixed Method
P17	Female	Academia	3rd year PhD	Qualitative Method
P18	Male	Academia	2nd year PhD	Mixed Method

### Interview protocol

Semi-structured interviews [70] were conducted virtually via Zoom video conferencing during July 2024. The consent was taken verbally from all the participants before the interview which is approved by the PennState IRB. Interviews lasted between 40 minutes - 1 hour, depending on the length of participant responses to interview questions. All interviews were recorded and transcripts for analysis were generated through transcription capabilities native to Zoom. We asked participants to share their primary research method, their understanding of open science practices, the conferences and journals in which they submit their research, their engagement in practices associated with open science, their familiarity with pre-registration, the importance of open science, ethical considerations to adopt these practices, support, and incentive needed to foster the open science culture.

### Data analysis

**Fig 1:**
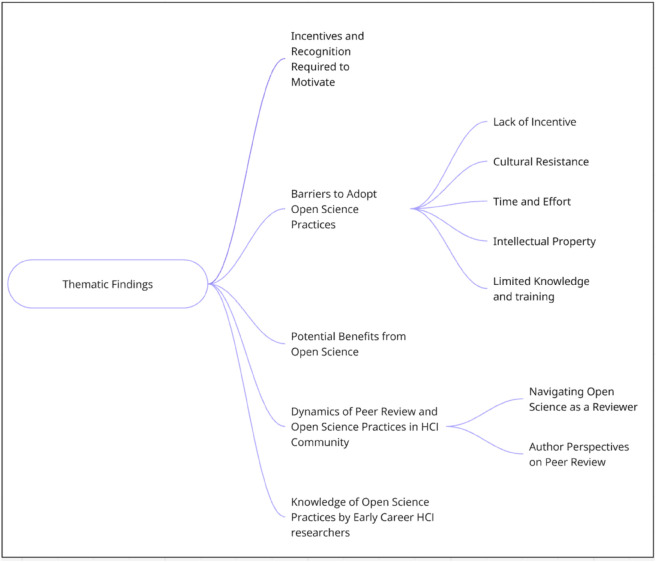
A visual representation of findings, themes, and sub-themes.

We analyzed the interview transcripts using thematic analysis as outlined by Blandford [71]. This qualitative data analysis method involves thoroughly reading the transcripts to identify patterns across the dataset and derive themes related to the research questions. We employed a collaborative and iterative coding process [72,73]. Initially, the first two authors read the interview transcripts multiple times to become familiar with the data. They independently coded all interview transcripts to identify initial tags and meaningful segments. After independent coding was completed, differences in coding were discussed and reconciled through collaborative review. Throughout the process, the first two authors met to discuss the meanings, similarities, and differences of identified themes and their relevance to the research questions. Once codes were aligned, the authors organized related tags and supporting quotes into thematic clusters. These clusters were iteratively refined into higher-order themes that reflected recurring patterns and deeper insights across the data. The author team made final decisions regarding the retention, removal, or reorganization of these themes collectively during weekly discussions. This process ensured analytical rigor by combining independent coding with systematic reconciliation and theme development grounded in the data. The coding process resulted in five themes, specifically, “knowledge of open science practices among early-career HCI researchers”, “dynamics of peer review and open science practices in the HCI community”, “potential benefits of open science”, “barriers to the adoption of open science practices”, and “incentives and recognition required to motivate”. Fig 1 represents a clear diagram of all the themes.

Our dataset is the code book generated by the first two authors of the paper after analysing the interview transcripts. The dataset contains 18 participants, labeled as P1 through P18. It contains all the themes that we got after the thematic analysis. We have shared quotes from each participant. The code book does not contain any private data provided by the participants. We have followed all the ethical guidelines provided by the Pennsylvania State University IRB. No personal or identifiable information was used.

### Ethical approval

Our study directly addresses research ethics and transparency. An explicit aim of our work is to seed more inclusive conversations around open science in HCI and highlight perspectives of early career HCI researchers. This study is not pre-registered. While we followed a detailed protocol and have tried so that there is no selection bias, preregistration might have strengthened the study’s methodological transparency and rigor. This is a semi-structured interview-based study. The study plan received an ethics waiver approved by the Pennsylvania State University’s Institutional Review Board (IRB) before starting data collection. The study number is STUDY00025251. Participants were fully informed about the nature of the study, potential risks, and their right to withdraw at any time without penalty before the study began. Verbal informed consent was obtained from all participants prior to the start of each interview. The consent process included a verbal explanation of the study’s goals, procedures, potential risks, participants’ rights (including the right to withdraw at any time), and measures taken to ensure confidentiality. Participants were given the opportunity to ask questions before agreeing to participate. Verbal informed consent was obtained from all participants prior to data collection. Participants were provided with a clear explanation of the study’s purpose, procedures, potential risks, and their rights, including the right to withdraw at any time without penalty. Consent was documented by the interviewer through a standardized script, and each interaction was audio recorded with participant permission as part of the consent process. The study protocol, including the verbal consent process. The IRB approved the use of verbal consent, and no third-party witness was required as per the guidelines provided in the approved protocol. We have only considered the responses where the participants have provided consent for the interview. Data was stored securely and only used for agreed-upon purposes. The collection and analysis of interview data is in compliance with all relevant institutional and legal guidelines for human subjects research.

## Findings

### Knowledge of open science practices by early-career HCI researchers

Here, we discuss participants’ background knowledge and attitudes toward open science practices. This helps us contextualize their perspectives on adopting open science practices. Some participants were well-informed about open science, others were only partially familiar with it. Notably, only one participant (P17) mentioned she was not at all familiar with open science. Although many researchers had some level of familiarity with open science, their understanding of these practices varied. For example, researcher P4 described open science primarily as an open-access archive, where papers are freely accessible.

*I guess, according to what I know, open science practice is basically making research available to the community without paywalls, or membership. So I would say, the open-access archive is probably the biggest open science thing that I know.*-P4

Other researchers, such as P2, P13, and P14, also viewed open science primarily as open access, similar to P4. P14 specifically highlighted the cultural differences in open science practices across different countries. He also emphasized the importance of equity that open access can provide to developing countries.

*My understanding, is that open science is a worldwide movement in order to push the boundaries of knowledge to the people of 3rd World countries, with very limited access. With this, they will be able to read through the research articles that everyone does. Basically bringing equity.*-P14

Interestingly, we found that participant P10 (qualitative researcher) believed that open science practices are relevant only to quantitative researchers—a view also shared by other researchers, such as P1, P15, P4, and P13. According to P10, data sharing applies exclusively to quantitative work.

*I am not so much familiar. But I have heard and read of things, but because I’ve not really done that much quantitative work I’m not familiar with the open science practices, I am on the qualitative side of things.*-P10

Despite the advancement of the open science movements and promoting best research practices, we found that researchers have very diverse perceptions of open science. For example, participant P11, a current PhD student, has a different understanding of open science practices compared to others. According to her, open science involves pre-registering and testing your hypothesis before publication, but she does not have proper clarity.

*I’m currently doing a survey and before the data collection we need to do the preregistration. We need to test the hypothesis that I have heard from my advisor. I have not done it before.*-P11

Familiarity with open science or open science practices often depends on researchers’ backgrounds, lab cultures, and collaborators. For example, P12 mentioned that he was introduced to these practices during his master’s studies while working at the university library. P2, P6, and P7 mentioned their advisors motivate them to adopt some of these practices in their research. P15, an early career UX researcher from the industry, noted that most funding bodies are funded by taxpayer money, and therefore the public should have access to the findings and research materials. However, she also believes that open science does not apply to qualitative research and mentioned that her company does not allow the sharing of such materials.

*I think open science makes your research available to others without being behind a paywall, allowing not only scientists but anyone to access and learn about the findings. Since funding often comes from the public, why shouldn’t the public, who have contributed to those funds, be able to see and use the research?*-P15

However, we observed P3, P6, P7, P8, P9, P16, and P18 are having a much better understanding of open science practices compared to the other participants.

*I know, this concept, and I know it is related to making your research publicly available. And so it can be more reproducible and transparent. Such as preregistering, like research plan, and publishing your data set and code.*-P18

### Peer review and open science practices in the HCI community

This section describes the culture of peer review in the HCI community through the HCI researchers. We observed that the majority had submitted to and reviewed for CHI, the leading HCI conference globally, with other conferences such as CSCW and CHI Play also being mentioned. Only a few researchers mentioned submitting to academic journals.

#### Author perspectives on peer review.

This section represents the open science practices of these researchers as authors during the submission of their papers. All of them who have been involved in interview studies mentioned that they have never shared interview transcripts, as doing so could reveal personal details. The transcripts contain a significant amount of sensitive information, and it is not a common practice to share. If they need to share those lots of time and effort are needed to anonymize those transcripts which does not carry any incentives according to P1. Additionally, the top conferences do not have any mandatory regulations to submit these documents, and reviewers do not typically ask for them mentioned by Participant P1. For the follow-up questions, she mentioned that she did not provide the codebook generated from the transcript and the interview protocols as well during submission.

*For the qualitative paper, I submitted to CHI as a 1st author for that one I didn’t provide any transcripts of my interviews. That’s the only data that I collected. And I guess recordings. But that’s not something that we would share because it would reveal the person’s identity. It’s not the common practice to share it. So it wasn’t something that was requested, reviewers didn’t even ask to see the transcripts.*-P1

Most HCI researchers do not consider the codebook as data that can be shared. However, we found that some researchers, like P2, P6, and P18, shared their interview protocols during conference paper submissions. P2 never shared her codebook. She also mentioned that she hasn’t seen others sharing it either. P2 further noted that when she first saw that CHI submissions required making the paper open-access, she thought it was a very positive step. She believes this is beneficial for researchers who cannot access papers due to paywalls. Although she supports these practices, she is also willing to share the final outcomes of her project as open-source software.

*For the CHI submission, they let you ask, like some additional documentation as supplemental. I’ve submitted my interview questions for that. But I haven’t submitted the codebook. I wasn’t sure but I don’t think I’ve seen other people do it before. However, I’m working on a project that will result in something like a system. And I intend to share it as open source through Github.*-P2

Not only P1 and P2, but all other researchers (Except P6 and P18) mentioned that they do not share their codebook for the qualitative research because they do not see how it would be useful to others. They also noted that if the top conferences do not ask to share the codebook, which means it is generally not considered important to do so. However, qualitative researchers like P4 expressed concerns about sharing her work before the acceptance of the paper.

*I feel like I wouldn’t be open to sharing protocols and my data until the papers have been accepted. Because I don’t want to make my process available to other people as it is my work.*-P4

Only, P6 and P18 mentioned that they have shared the codebook with the submission to provide more transparency and validity. They also believe that this can improve their acceptance of the paper. P5, a mixed method researcher mentioned they always try to share their code and data.

*Yes, we have shared our code. We made the prototype public and we shared it publicly. The URL was public. We made it like a web-based prototype. So anyone can go to the URL and use that prototype to program.*-P5

Whereas, another mixed method researcher P8, mentioned she did not share the code because it was a prototype and it is not a common practice or asked to share these things. But for some journal submissions when it is mandatory to share the code and data, people share more mentioned by P16 and P8 because that is required to get the publication done.

*The study during my PhD, we didn’t share because we developed a prototype. We didn’t make that code available and then the data for the interviews, I don’t believe we made that available either.*-P8

Participants P1, P2, P4, P10, and P17 also mentioned that for the conference submission, they were not asked by the reviewers to share the code, data, or protocols. It is more about how you have described the method section clearly for the readers. The industry UX researcher P15 mentioned not sharing the data because the data is for the company and the company does not agree to share openly. However when we had a discussion with P3, another industry researcher mentioned data sharing, code sharing, and positive support for open science practices.

*The product design that I did last year for CHI submission. We interviewed a bunch of product designers. I didn’t share that, because that was like kind of internal data for the Company.*-P15

#### Navigating open science as a reviewer.

During the interview study, we also asked participants to share their experiences as a reviewer. The goal was to understand do they look for the supplemental materials submitted by the authors for the paper, and the importance of open sharing for peer review. The majority of the participants except P4, P6, P7, P9, and P12 have reviewed HCI papers. The majority of the participants have reviewed for CHI, CSCW, and some other NLP conferences. It was observed that the authors weren’t asked by the participants to share code or data as it is not mandatory by the conferences. The participants try to evaluate the method section carefully to understand the clarity if that is not clear then they ask clarification questions as mentioned by P1.

*I have reviewed CSCW and CHI, but I never asked the authors to submit the data, codebook, or interview protocols. I feel like if the conference is not asking then why should I ask to share? I focus on the methods section if that has been explained clearly or not. If the method section is not clear then I ask my clarification questions.*-P1

The same responses were observed from the majority of the participants(P2, P3, P8, P10, P11, P17, P18). If it is not mandatory to share by the conferences then they usually don’t ask to share during the review. Exceptionally, participant P5 mentioned that he has reviewed one such paper where they did not mention the parameters clearly which they have asked during the interviews. Therefore he asked to share the interview questions to have more clarity.

*I also reviewed one such paper, where the authors have used different parameters during the interview but they haven’t mentioned that clearly in the method section. Therefore I asked them to share the interview questions. If that’s not there, it’s very difficult to understand.*-P5

Participant P16, assistant professor of HCI, mentioned that is very important to share the protocols without which you cannot have the whole understanding of the method. She also mentioned that some of the papers share very unique methods but they don’t share the protocols therefore other researchers can not implement them correctly. A very unique fact she shared about her experience as a reviewer for the CHI. Many authors started their experimental protocols during the review process for the CHI conference. But they drop the supplemental during the camera-ready version.

*I have experience as a reviewer during the process of CHI, and lots of authors have started submitting the protocols. But during the camera-ready version, they just drop the supplemental.*-P16

Participant P7 mentioned clearly that in her lab the other researchers are from NLP, AI, and machine learning. Many of those AI and ML conferences have mandatory rules for data. code and materials sharing. Therefore researchers share more to get their paper published. From the observations, it is very clear that participants are really not very comfortable asking the authors of the papers during the peer review to share any data, code, or other materials as it is not mandatory by the conference itself. Especially the early career researchers when they have very little experience and they don’t know how to ask these questions as they are also not involved that much in open sharing mentioned by P10.

### Benefits of open science

The majority of the participants think open science is helpful in many ways. We observed there are few researchers who think there is no proper benefit of open science practices in HCI. For example, P4, a PhD candidate, mentioned during the interview that she doesn’t know how open science can be beneficial for qualitative researchers like her. She also mentioned that it is more important for quantitative researchers. However, some of them have benefited from open science. For example, mentioned by Participant P1, as she is a qualitative researcher, does not have experience in survey design. For one of her studies, she had to do a survey she looked for open survey data sets and how the other researchers conducted the survey design. Many of the researchers have put their whole survey in the appendix with other materials. This helps her to design her study.

*I was building a survey for my study. I did look for published papers that had the data set or like, had those appendices on, how they conducted the survey, and their survey design. They usually put their whole survey in the Appendix. That helped me build my own survey because I had never done it before.*-P1

According to P6, a PhD candidate, open science practices can increase the citation count of the papers and also increase the chances of acceptance as the reviewers will feel the work is validated and rigorous. Therefore he is very positive about open science practices.

*I’m all for sharing materials. Because number one, it increases the probability of acceptance. Because of the rigor of the paper right where a reviewer is reviewing it. They will feel more confident about the paper as it’s transparent. Also in the future, it will help us to get more citation counts as well.*-P6

We found open source data is another thing that is helping many researchers. For example, participant P13 mentioned how open-source data sharing is helping her during the PhD journey.

*For my research, I am using some clinical data that data was shared by others. That is some kind of open-source data which I am using for my research.*-P13

On the other hand, P11, a mixed method researcher, mentioned how she is using open-source code for her research which saves a lot more time than doing the same thing that already exists. Rather that time can be used to build something different than the existing work she mentioned.

*I am using many open-source codes provided by the other researchers. This makes my research faster and I don’t have to waste my time on what already exists in literature. Rather I can invest in something new.*-P11

The main benefits of open science mentioned by the participants are transparency and validity of research practices. It is ethical to ask to have transparent research mentioned by P17. These are the basic practices of good research and these increase the reproducibility and replication mentioned by P18. However, he mentioned that reproducibility is not for qualitative researchers; rather, this is for quantitative research. Due to the increased use of technology and large language models, like ChatGPT, it is very easy to fake data and make fake research mentioned by P3, P5, P6, and P7. Open science practices can improve the research quality.

*I feel people can also fake data. And it’s very easy nowadays, right? Because of chatGPT and other tools like that. You can always create a normal-looking research but which is fake.*-P3

A very important point has been mentioned by P14, an international PhD student, the importance of open sharing of research papers in the global south mainly for those universities that can not pay for access to publications like IEEE or ACM. Open science has opened the doors for them at least to access a lot of research papers that were completely impossible or they had to use pirated versions in the past.

*The university where I got my bachelor’s and master’s degree back in my home country, did not have any subscription services to access research papers. We could not afford to buy a single piece of paper for 10 dollar. I know it’s not a good way to describe it. But we unfortunately had to pirate the papers, because there was essentially no other way for us.*-P14

### Barriers to adopt open science practices

This section represents all the barriers mentioned by the participants to adopting open science practices in their research. Table 2 represents the summary of all these barriers.

**Table 2:** Barriers to the adoption of open science practices in HCI.

Barriers	Description
Lack of Incentive	No formal rewards for engaging in open science; efforts often go unrecognized.
Cultural Resistance	Norms in HCI discourage open sharing; fear of criticism from peers and reviewers.
Limited Knowledge and Training	Researchers lack structured training in open science practices and tools.
Time and Effort	Preparing and anonymizing data for sharing is time-intensive and undervalued.
Intellectual Property Concerns	Fear of being scooped or losing control over novel ideas and data.
Lack of Incentives and Recognition	Need for clear benefits, awards, or mandates from conferences and institutions.

#### Lack of incentive.

Academic reward systems in the HCI community often prioritize publications and citations over open practices, leading researchers to focus on these metrics rather than sharing data, code, methods, or any other materials mentioned by all the participants. Highlighted by all the participants that currently there is no incentive to do open science practices which needs more effort and time. Also, the industry participants feel the same. Some researchers are highly motivated by themselves for example P6 but that number is very rare. Currently, it completely depends on personal ethics and self-motivation as mentioned by P8.

*Currently there is no incentive for open science practices, it is all about self-motivation, ethics, and discipline.*-P8

#### Cultural resistance.

Many scientific communities are motivated by traditional practices of closed research, where sharing data, code, protocols, and methods is not the norm. The majority of these participants mentioned that in HCI open sharing and open science practices are not common. P1 and P17 are concerned that openly sharing data or methodologies will lead to more criticism or negative scrutiny during the peer review process.

*I am scared to share the interview protocols because I feel this can create more criticism by the reviewers. Open science practices are not a common practice in the HCI community.*-P17

The primary focus of all participants is clear: they aim to get their papers published. Their priority is to publish in top HCI conferences, such as CHI. For these conferences, it is not required to submit data and code, which diminishes the emphasis on open science practices. When is it not required that implies it is not that important to submit according to the majority of the participants except P2. We observed these practices are very culturally diverse depending on the lab culture or the research community. For example, P17 added that last year 2023 their lab group started talking about all these practices.

*From last year I remember our lab members and my advisors started to talk about data sharing in the CHI and CSCW communities. I heard that researchers started to emphasize the importance of sharing their interview protocols and other materials in the supplemental.*-P17

On the other hand side, P7 mentioned that her co-authors don’t support open sharing which highlights the traditional lab culture.

*My co-authors mentioned to me that the norm in the field of HCI is not to share qualitative data. If we ask participants that we are going to share all of their data, like everything that they say in the interview, in conferences or journals. They might not be very transparent with us,*-P7

#### Limited knowledge and training.

We observed from this interview study that researchers lack the necessary training and support to implement open science practices effectively, from data management, data sharing, and protocol sharing to navigating open-access publication. For example, Participant P10 has mentioned clearly that she doesn’t know how to share the codebook. The codebook has different levels of coding and which one should be shared is not clear to her. Also, P2 mentioned that most of the time codebooks are not very well structured which can be shared. The semi-structured interviews, do not always have the exact questions in the protocols, they always go with the flow and explore more insights. P1 and P10 mentioned that they don’t know in which structure they should share these protocols as they are semi-structured. Also, participants are not trained to anonymize the transcripts.

*We did rounds of open coding and deliberation. Then we sort of like focus those together. So we did some rounds of focus coding. And finally, we divided all of these relevant codes into the 3 themes, that sort of answer our research questions. So I’m just trying to wonder, at what stage would the code book require to be shared? I’m not sure if I know of the methodology, for at least semi-structured interviews to share transcripts and the protocols.*-P10

It was very interesting to observe that Participant P4, a first-year PhD student in HCI, doesn’t know where to share these materials. After more in-depth conversations she mentioned that she never thought the supplemental is for data sharing or protocol sharing.

*At least for the papers that I have submitted to the CHI conference, there isn’t really like on Pcs. I haven’t really seen anything to make these kinds of submissions for open science practices.*-P4

A significant number of researchers report having no formal training or attending workshops related to open science practices. The lack of structured training is a major barrier to adoption. Some participants like P1, P5, P7, P11, and P12 mentioned that even when training was available, it was very basic and lacked depth, particularly in areas like reproducibility, and transparency. There is a clear need for more formalized, detailed training programs on open science practices mentioned by the participants. This training should be integrated into existing curricula and made available to researchers at all career stages, especially early-career researchers. Participants suggest that such training would be particularly useful if it provides practical guidance on how to implement open science practices in their specific research areas.

#### Time and effort.

Preparing data and methods for open sharing requires a significant amount of time and effort which itself is very difficult for early career researchers. The majority of these participants like P1, P5, P8, and P11 mentioned that they do not have that much time to invest in open science practices. For example, P11 mentioned that she does not have time to manually go through all transcripts and anonymize them. And, if she is going to invest that much time and effort then she would expect some incentive for that which is not currently afforded in the academic system.

*I don’t have time to manually go over every transcript and make them anonymized. It will take lots of effort without any incentives. It is not required in this community.*-P11

For qualitative research, sharing transcripts is a major challenge. It involves considerable personal data and, at the same time, it is not well structured as it is automatically coded. In addition, researchers do not always code the entire transcript as it would take considerable time.

*If I have to share the transcripts then I have to fix the whole transcript so it’s very time-consuming. I only fix the relevant parts that are needed. Also if no one is doing that in your community then why should I invest that much time which is not cost-effective.*-P1

#### Intellectual property.

Concerns about losing control over data and intellectual property can make researchers reluctant to share their work openly which was observed from the conversations with P4, P8, and P14. These three participants are very scared that their ideas and data can be scooped or stolen by others. According to the P4, she is not comfortable with the sharing because it is her own research and she wants to continue doing based on her findings in the future. P8, the postdoctoral researcher also thinks in the same way and mentions her concern about the protection of intellectual property.

*I think the only reason to withhold sharing is the risk for sharing, and I think that my biggest concern with open science practices is, where are the protections for intellectual property? I think that there’s like a novelty aspect to HCI research where the most novel ideas gain the most attraction. And when you share your research, and then someone else goes and uses what you’ve built, then it reduces the novelty of your subsequent work.*-P8

### Incentives and recognition required to motivate

One of the significant barriers to open science practices is that there is no incentive for researchers. Researchers need a clear understanding of the benefits associated with adopting open science practices as mentioned by P1. She added a need for cost-benefit analysis which justifies the additional time and effort required to make data, code, and other materials openly available. Many researchers like P4, and P8 currently see no incentive or direct benefit from sharing their data, which discourages them from engaging in open science. Conferences and academic institutions could incentivize open science by offering awards for papers that share supplementary materials or demonstrate reproducibility mentioned by P3.

*I think conferences can provide different awards or recognition for papers that share more supplementary materials, and which are in general very open and reproducible, that will be a good way to encourage and recognize their efforts.*-P3

This recognition could encourage more researchers to engage in open science practices. The adoption of open science practices would be more widespread if major conferences and journals required or strongly encouraged some of these practices. For instance, if leading conferences in the HCI field, such as CHI, mandated open science practices, it could set a standard that others would follow, as mentioned by P7.

*I think that it may be beneficial if the major conferences like CHI require you to practice open science, then most HCI researchers will be inclined to do that, a lot of the conferences in machine learning started doing that when a lot of papers came out which mentioned that they are not being able to replicate.*-P7

### Discussion

We observe a diverse yet often incomplete understanding of open science practices among early-career HCI practitioners. The majority do not have an accurate and complete understanding of open science. Some researchers perceive open science as being primarily relevant to quantitative research, with less applicability to qualitative research methods. Other participants conflated open science with open publishing practices solely [74]. A significant number of participants report limited knowledge and training in open science practices, particularly how to effectively share data, protocols, and other research materials. Researchers are unsure how or where to share semi-structured data, especially when anonymization is hard. Researchers fear criticism, loss of novelty, or misuse of data. We also found that Lab culture and advisors have a strong influence. Cultural resistance and lack of clear incentives emerge as major barriers to the advancement of open science practices. Our findings bring to light the critical role of conferences in driving these practices. Participants indicated that if major HCI conferences strongly encouraged open science practices, this would set a standard for the field and likely increase participation. Table 3 represents the summary of all recommendations provided.

**Table 3:** Summary of recommendations on open science practices in HCI.

Recommendations	M ain Points
Encourage a Cultural Shift Towards Openness	- Senior researchers should model and advocate - Early-career researchers need mentorship - Create inclusive discussions - Emphasize cultural change in labs
Enhance Training and Education	- Integrate OS training into curricula - Educate on open licenses - Offer institutional workshops - Promote institutional adoption of OS
Role of Conferences and Journals	- Journals should promote OS. - Conferences should strongly encourage OS. - Mandate sharing of protocols and codebooks - Provide guidelines and webinars to support researchers
Incentivize Open Science Practices	- Institutions should recognize for promotion - Awards and recognition for open practices. - Develop metrics to credit data/code sharing
Intellectual Property Concerns	- Use appropriate licenses - Repositories like Zenodo, OSF, GitHub support
Potential Burdens of Open Science Practices	- Recognize the added burden on qualitative researchers. - Concerns include misuse, criticism, labor intensity - Offer flexible guidelines - Suggest alternatives like sharing codebooks instead of full transcripts.

In addition to identifying challenges, our findings highlight several encouraging developments around open science in the HCI community. Many early-career researchers expressed growing awareness of open science practices such as data sharing, preregistration, and transparent peer review, even if they had not yet implemented them. Some participants described how peer review processes, particularly at venues like CHI, were beginning to incentivize openness through reviewer expectations and artifact submission tracks. Others pointed to tangible benefits of engaging in open science, including increased visibility of their work, increased diversity, opportunities for collaboration, and a stronger sense of research credibility. These positive perspectives suggest that while structural and cultural barriers remain, the groundwork for broader adoption of open science in HCI is already taking shape. Finally, we provide recommendations for the stakeholders across the scientific landscape in HCI to integrate these practices into the community.

#### Encourage a cultural shift towards openness.

Cultural resistance to open science practices is deeply rooted in the HCI community, with many researchers adhering to traditional norms of closed research [75]. To foster a cultural shift towards openness, senior researchers and leaders within the HCI community should advocate for open science practices, setting an example for others to follow. It’s also important to recognize that early-career researchers may feel vulnerable when practicing openness in communities where it is not yet standard. Senior scholars can mitigate this by mentoring and advocating on behalf of junior colleagues. By publicly endorsing and practicing open science, these leaders can influence the broader community and help normalize these practices. Organize discussions, panels, and forums within the HCI community to engage researchers in conversations about the benefits and challenges of open science. These events should be inclusive, encouraging participation from researchers at all career stages and across different subfields of HCI. Establish peer support networks where researchers can share their experiences, challenges, and successes with open science practices. These networks can provide a platform for exchanging ideas and strategies, helping to build a community of practice around open science. The main obstacles to change are not technical or financial but social. Although scientists tend to maintain the status quo, they are also the ones who can drive change. By embedding open science into the values and everyday practices of the HCI community, we can create a more inclusive, transparent, and impactful research culture for future generations.

#### Enhance training and education.

There is a clear need for more formalized training programs that are integrated into the educational curriculum for HCI researchers. These programs should be comprehensive, covering the practical aspects of open science, including data management, ethical considerations, and the use of open-access platforms [76,77]. This should cover how the researchers can share protocols and anonymize qualitative data. Such training would be especially beneficial for early-career researchers, who are still forming their research practices and could be more easily guided toward a culture of openness. Researchers from psychology tailored support targeting early-career researchers and underrepresented regions to ensure equity in open science access and practice [78]. Understanding open licenses, such as Creative Commons (CC), should also be part of open science education. These licenses empower researchers to control the use of their work while promoting reuse and collaboration. By strategically using open licenses, patents, defensive publications, and collaborative models, researchers and institutions can protect their intellectual property while contributing to the collective advancement of science.

Academic institutions and professional organizations should regularly offer workshops and seminars focused on the practical aspects and training of implementing open science practices for undergraduate and graduate students [79]. Many academic institutions have research policies or codes of practice that clarify the principles, ethical foundations, and expectations for researchers’ behavior within the institution. However, open science policies, such as those related to open access and open data, are seldom included in these research policies [80]. Some institutions have begun developing, adopting, and implementing open science policies, primarily over the past few years [81]. Furthermore, the majority of conferences offer “Course” sessions as part of their registration, aimed at helping early-career researchers learn about emerging topics in the field. These sessions could also serve as an effective platform for disseminating knowledge and fostering discussions on open science practices.

#### Role of conferences and journals.

The findings also suggest that the role of conferences and journals is pivotal in driving the adoption of open science practices. To support researchers in improving transparency and rigor, several journals have begun adopting and promoting standardized reporting frameworks such as the JARS-QUANT (Journal Article Reporting Standards for Quantitative Research) [82] and JARS-QUAL (for Qualitative Research) [83] guidelines developed by the American Psychological Association (APA). These frameworks provide detailed checklists to guide authors in reporting essential methodological details, thereby fostering reproducibility and clarity. Researchers can access these guidelines through the APA website, and many journals now require or recommend their use during the submission process. However, participants indicated that if major HCI conferences like CHI and CSCW mandated or strongly encouraged open science practices, this would set a standard for the field and likely increase participation, as HCI is highly conference-focused. We understand there can be potential barriers to mandating these practices in the conferences. HCI conferences, such as CHI and CSCW, should consider making some of the open science practices a requirement for submission. This could include mandatory interview protocol sharing for qualitative works, codebook sharing, pre-registration of hypothesis-driven studies, and code and data sharing for quantitative research, which are publicly funded projects. If the researchers are not able to share, then they should provide a proper justification for not sharing. Conferences could provide clear guidelines on what is expected and offer support to researchers unfamiliar with these processes through some webinars. Journals in the HCI field should also consider integrating open science guidelines [58] for the submission of supplementary materials, such as data and code, as part of the publication process. At the same time, we think pre-registration for qualitative or exploratory works should not be mandatory for these journals.

#### Incentivize open science practices.

One of the most significant barriers to adopting open science practices identified in this study is the lack of clear and tangible incentives. Academic institutions should consider integrating open science contributions into the criteria for promotions, selection, and tenure [84]. By recognizing and rewarding efforts to share data, code, and research protocols openly, institutions can create a powerful incentive for researchers to adopt these practices [85,86]. Leading HCI conferences and journals should introduce specific recognition for papers that excel in open science practices. For example, awards could be given for best practices in data sharing, reproducibility, or the most comprehensive supplementary materials. Such recognition would not only motivate researchers but also set a benchmark for quality in science. Encourage the development and use of metrics that recognize the impact of open science practices [87,88], such as citations of datasets, code, and other shared materials. These metrics could be integrated into existing impact assessments, helping researchers see the tangible benefits of their open contributions.

#### Intellectual property concerns.

While concerns around intellectual property (IP) were frequently cited by participants, particularly among those developing novel qualitative frameworks or prototypes, there are established mechanisms to help researchers share their work openly while protecting ownership. For instance, researchers can apply Creative Commons (CC) [89] licenses to datasets, codebooks, and protocols to specify how others may use, attribute, or build upon their work. While CC licenses are ideal for data, codebooks, and publications, open-source software should use licenses specifically designed for code, for example Massachusetts Institute of Technology License (MIT), and the GNU General Public License (GPL), which clarify usage, modification, and distribution rights. These licenses offer a spectrum of protections, enabling researchers to maintain credit while promoting reuse. Moreover, data and code repositories, for example, Zenodo, OSF, and GitHub, now support formal citation mechanisms, ensuring that shared materials can be acknowledged in scholarly metrics and tenure evaluations. Including this kind of structured guidance in HCI training and conference documentation could help normalize open sharing practices, especially among early-career researchers [90].

#### Burden of open science practices.

We also acknowledge that open science practices may increase the burden on qualitative researchers, especially those working with sensitive or vulnerable group data. The findings reveal that many participants expressed concerns about the added workload and a misaligned reward structure. Several viewed these practices as burdensome, with little to no perceived benefit. As Participant 8 noted, there is apprehension about the risk of others misusing or appropriating their data for future studies. Others worried that making their data, code, and protocols openly available could invite undue criticism or amplify perceived limitations in their work. Some also cited practical barriers, such as a lack of support from co-authors, which further hinders implementation. This again brings us to highlight further how a shift in community mindset and a top-down approach of leading by example may be required. With qualitative research, there are existing challenges to publications, and open-sharing of the data and protocol may create additional pressure on researchers, making it harder for them to publish and share their findings [37,91]. For example, anonymizing semi-structured interview transcripts is labor-intensive, and the process is not always straightforward due to contextual nuances. As several participants noted, sharing these materials may also violate participant trust or compromise future research plans. Sometimes, before an interview, if you mention that the data will be shared, then participants may not open up properly as well. There should be flexible guidelines coupled with the option to submit ethical justifications for non-disclosure can uphold transparency while respecting methodological diversity and ethical complexity. Also, they can share the codebook instead of the transcripts, which can make the process easy for them.

## Potential biases

As our study is self-reported, interview-based research, we acknowledge that our findings may be influenced by recall bias and social desirability bias. Participants may have unintentionally overreported behaviors aligned with community norms or values, such as openness, collaboration, or ethical sensitivity, particularly in response to questions about practices they perceive as socially or professionally desirable. Although we encouraged reflective and candid responses, these biases remain a consideration in interpreting the findings. To strengthen validity and build on this work, future research could triangulate interview data with behavioral evidence such as actual rates of artifact sharing (e.g., data, code, or preprints), public version control activity, or citation networks—providing complementary insights into how values and practices manifest in HCI research workflows.

## Limitations and future work

While our study provides important and valuable insights into the perceptions and challenges of adopting open science practices within the HCI community, several limitations should be acknowledged. The study is based on semi-structured interviews with 18 early-career HCI researchers, which, while providing in-depth qualitative data, may not be fully representative of the broader HCI community. This study focuses exclusively on early-career HCI researchers based in the United States, which may limit the generalizability of the findings to other geographic or cultural contexts. Academic norms, funding structures, and institutional pressures can vary significantly across countries, potentially shaping researchers’ experiences in different ways. Future research should explore cross-cultural perspectives to better understand how regional, institutional, and cultural factors influence early-career trajectories and challenges in HCI. This paragraph has been added to the limitation section. However, the main intention was to capture the perceptions from the early career researchers. The perspectives captured in this study may vary from those of other researchers, particularly in different geographic regions or sub-fields of HCI. Our study primarily focuses on qualitative insights, exploring the attitudes, experiences, and perceived challenges related to open science practices. While this approach provides rich, contextual understanding, it does not offer quantitative measures of how widespread these attitudes and practices are within the HCI community. Future research could complement our findings with quantitative surveys to assess the prevalence and variability of these practices across a larger and more diverse sample.

## Conclusion

Our research reveals that while there is a growing awareness of open science within the HCI community, its implementation remains unequal with different barriers. One of the main reasons for this is that researchers exhibit a wide range of understanding about open science practices. Key barriers to adoption include the lack of clear incentives and rewards, cultural resistance within the HCI community, limited training opportunities, concerns about intellectual property, limited time, and ethical concerns. These challenges are compounded by an academic culture that prioritizes traditional research outputs, such as publications and citations, over the principles of transparency and reproducibility central to open science. To advance the adoption of open science, our study recommends the development of institutional and conference-level incentives, the standardization of open science requirements, and the integration of comprehensive training programs into the education of HCI researchers.
