# Supplementary material for: Open science practices among early-career human-computer interaction researchers in the US
Source: PLoS One. 2025 Nov 12;20(11):e0334692. doi: 10.1371/journal.pone.0334692 (PMC12611103; doi:10.1371/journal.pone.0334692)
Supplement: Attachment — Submitted filename: PONE-D-25-11453 rebuttal.pdf [file pone.0334692.s002.pdf]

PONE-D-25-11453
Embracing Transparency: A Study of Open Science Practices Among Early Career HCI Researchers
PLOS ONE

Dear Dr. Chakravorti,

Thank you for submitting your manuscript to PLOS ONE. **After careful consideration, we feel that it has merit but does not fully meet PLOS ONE's publication criteria as it currently stands.** Therefore, we invite you to submit a revised version of the manuscript that addresses the points raised during the review process.

Please submit your revised manuscript by Aug 07 2025 11:59PM. If you will need more time than this to complete your revisions, please reply to this message or contact the journal office at plosone@plos.org. When you're ready to submit your revision, log on to https://www.editorialmanager.com/pone/ and select the 'Submissions Needing Revision' folder to locate your manuscript file.

Please include the following items when submitting your revised manuscript: (Documents needed to be upload)

- A rebuttal letter that responds to each point raised by the academic editor and reviewer(s). You should upload this letter as a separate file labeled 'Response to Reviewers'.
- A marked-up copy of your manuscript that highlights changes made to the original version. You should upload this as a separate file labeled 'Revised Manuscript with Track Changes'.
- An unmarked version of your revised paper without tracked changes. You should upload this as a separate file labeled 'Manuscript'.

If you would like to make changes to your financial disclosure, please include your updated statement in your cover letter. Guidelines for resubmitting your figure files are available below the reviewer comments at the end of this letter.

If applicable, we recommend that you deposit your laboratory protocols in protocols.io to enhance the reproducibility of your results. Protocols.io assigns your protocol its own identifier (DOI) so that it can be cited independently in the future. For instructions see: https://journals.plos.org/plosone/s/submission-guidelines#loc-laboratory-protocols. Additionally, PLOS ONE offers an option for publishing peer-reviewed Lab Protocol articles, which describe protocols hosted on protocols.io. Read more information on sharing protocols at https://plos.org/protocols?utm_medium=editorial-email&utm_source=authorletters&utm_campaign=protocols.

We look forward to receiving your revised manuscript.

Kind regards,

Mu-Hsuan Huang
Academic Editor
PLOS ONE

Journal Requirements:

When submitting your revision, we need you to address these additional requirements.

1. Please ensure that your manuscript meets PLOS ONE's style requirements, including those for file naming. The PLOS ONE style templates can be found at

https://journals.plos.org/plosone/s/file?id=wjVg/PLOSOne_formatting_sample_main_body.pdf and

https://journals.plos.org/plosone/s/file?id=ba62/PLOSOne_formatting_sample_title_authors_affiliations.pdf

Response: Thanks for the feedback. We have checked and the manuscript meets PLOS ONE's style format.

2. In your Methods section, please include additional information about your dataset and ensure that you have included a statement specifying whether the collection and analysis method complied with the terms and conditions for the source of the data.

Response: All the statements specifying the collection and analysis method complied with the terms and conditions for the source of the data are present in the Ethical approval section under Methodology. We have added more details about the dataset in the Methodology section.

3. In the ethics statement in the Methods, you have specified that verbal consent was obtained. Please provide additional details regarding how this consent was documented and witnessed, and state whether this was approved by the IRB.

Response: Thank you for the suggestion. Details about the consent have been clearly described in the Ethical Approval section under Methodology.

4. PLOS requires an ORCID iD for the corresponding author in Editorial Manager on papers submitted after December 6th, 2016. Please ensure that you have an ORCID iD and that it is validated in Editorial Manager. To do this, go to 'Update my Information' (in the upper left-hand corner of the main menu), and click on the Fetch/Validate link next to the ORCID field. This will take you to the ORCID site and allow you to create a new iD or authenticate a pre-existing iD in Editorial Manager.

Response: Tatiana Chakravorti : 0000-0002-4986-4921

5. Thank you for uploading your study's underlying data set. Unfortunately, the repository you have noted in your Data Availability statement does not qualify as an acceptable data repository according to PLOS's standards.

At this time, please upload the minimal data set necessary to replicate your study's findings to a stable, public repository (such as figshare or Dryad) and provide us with the relevant URLs, DOIs, or accession numbers that may be used to access these data. For a list of recommended repositories and additional information on PLOS standards for data deposition, please see https://journals.plos.org/plosone/s/recommended-repositories.

Response: We have uploaded the data using Github which is allowed. If that is not accepted let us know. We will again upload it using some other platform.

6. Please amend the manuscript submission data (via Edit Submission) to include author Sanjana Gautam.

7. Please amend your authorship list in your manuscript file to include author Sanjana Gautam.

Response: Author Sanjana Gautam has been added.

8. We note you have included a table to which you do not refer in the text of your manuscript. Please ensure that you refer to Table 1 in your text; if accepted, production will need this reference to link the reader to the Table.

Response: Table 1 has been referred to in the text.

[Note: HTML markup is below. Please do not edit.]

Reviewers' comments:

Reviewer's Responses to Questions

Comments to the Author

1. Is the manuscript technically sound, and do the data support the conclusions?

The manuscript must describe a technically sound piece of scientific research with data that supports the conclusions. Experiments must have been conducted rigorously, with appropriate controls, replication, and sample sizes. The conclusions must be drawn appropriately based on the data presented.

Reviewer #1: Partly

Reviewer #2: Partly

---

2. Has the statistical analysis been performed appropriately and rigorously?

Reviewer #1: Yes

Reviewer #2: I Don't Know

---

3. Have the authors made all data underlying the findings in their manuscript fully available?

The PLOS Data policy requires authors to make all data underlying the findings described in their manuscript fully available without restriction, with rare exception (please refer to the Data Availability Statement in the manuscript PDF file). The data should be provided as part of the manuscript or its supporting information, or deposited to a public repository. For example, in addition to summary statistics, the data points behind means, medians and variance measures should be available. If there are restrictions on publicly sharing data—e.g. participant privacy or use of data from a third party—those must be specified.

Reviewer #1: Yes

Reviewer #2: Yes

---

4. Is the manuscript presented in an intelligible fashion and written in standard English?

PLOS ONE does not copyedit accepted manuscripts, so the language in submitted

articles must be clear, correct, and unambiguous. Any typographical or grammatical errors should be corrected at revision, so please note any specific errors here.

Reviewer #1: Yes

Reviewer #2: No

---

5. Review Comments to the Author

Please use the space provided to explain your answers to the questions above. You may also include additional comments for the author, including concerns about dual publication, research ethics, or publication ethics. (Please upload your review as an attachment if it exceeds 20,000 characters)

Reviewer #1: The manuscript provides a timely qualitative exploration of open science adoption barriers and perceptions within the HCI community. The study is well-motivated, methodologically sound, and offers actionable recommendations. However, some areas require clarification or improvement.

Strengths:
1. **Relevance**: Addresses a critical gap in understanding open science practices among early-career HCI researchers, aligning with broader reproducibility debates.
2. **Methodology**: Robust use of semi-structured interviews (n=18) and thematic analysis, with clear documentation (e.g., GitHub repository for protocols).
3. **Findings**: Identifies key barriers (e.g., lack of incentives, cultural resistance) and benefits (e.g., transparency, citations) with nuanced qualitative insights.
4. **Recommendations**: Practical suggestions (e.g., cultural shifts, conference mandates) are well-grounded in data.

Weaknesses and Suggestions for Improvement:
1. **Sample Limitations**:
- The study's focus on U.S.-based researchers limits generalizability. Explicitly acknowledge this and recommend future cross-cultural studies.
- Consider adding demographic details (e.g., institutional types, subfields) to contextualize diversity within the sample.

Response: Thank you for the suggestion. We have addressed the "U.S.-based researchers limits generalizability" in the limitation section of the paper.

We have added the number of institutional types (like R1 or R2 institutes) in the participant recruitment section and the year of experience for all the researchers in Table 1. We have added their research subfields in the dataset.

2. **Self-Report Bias**:
- While noted in the abstract, emphasize how recall/social desirability biases might affect findings (e.g., overreporting openness). Suggest triangulation with behavioral data (e.g., artifact sharing rates). —(Both)

Response: Thank you for the suggestion. As our study is self-reported, interview-based research, our findings may be influenced by recall bias and social desirability bias. We have added a section for this as recommended after the Discussion and the section is called “Potential Biases”. The “Recommendation” section has been named “Discussion” as suggested by R2.

3. **Thematic Analysis**:
- Clarify how inter-coder reliability was ensured during theme development. A brief description of the reconciliation process would strengthen rigor.

Response: Inter coder reliability has been described in the data analysis section under Methodology as suggested.

4. **Recommendations**:
- Expand on how to address intellectual property concerns (e.g., Creative Commons licensing examples).
- Discuss potential downsides of mandating practices (e.g., burden on qualitative researchers).

Response: Thank you for the suggestion. We have added two sections one is for the potential downsides of mandating these practices which is called “Potential Burdens of Open Science Practices” and the other one is to address the intellectual property concerns which is named as “intellectual property concerns and remedies”. Both the sections have been added under “Discussion”.

5. **Structural Notes**:
- The "Related Work" section could better integrate HCI-specific challenges (e.g., qualitative vs. quantitative transparency).
- Tables/visualizations (e.g., a summary of barriers/themes) would improve readability.

Response: Thank you for the suggestion. We have added a section called “Challenges in Qualitative and Quantitative Research for Open Science” in the related work section to address qualitative vs quantitative challenges. We have added Figure 1 to represent the

visualisation for the themes. We have added Table 2 which represent different barriers and Table 3 which represent the summary of recommendations to improve readability.

5. **Literature review**:
The manuscript should make greater use of other sources and studies that have addressed related topics. The following articles are among the relevant articles that need to be cited and used in this manuscript:
- Zarghani, Maryam; Nemati-Anaraki; Leila, Sedghi, Shahram; Noroozi Chakoli, Abdolreza; Rowhani-Farid, Anisa. (2023). "The Application of Open Science Potentials in Research Processes: A Comprehensive Literature Review". Libri. DOI: 10.1515/libri-2022-0007
- Zarghani, Maryam; Nemati-Anaraki, Leila; Sedghi, Shahram; Noroozi Chakoli, Abdolreza; Rowhani-Farid, Ansia. (2023). ”Iranian researchers’ perspective about concept and effect of open science on research publication”. BMC Health Services Research. 23, 437. DOI: 10.1186/s12913-023-09420-9

Response: Thank you for pointing out these references. All these articles have been studied and cited properly in the related work section.

Ethical and Data Considerations:
- Ethics approval (IRB) and consent procedures are clearly documented.
- Data availability is commendable (GitHub), but ensure all links are functional at publication.

Conclusion:
This manuscript makes a valuable contribution to HCI and open science literature. With minor revisions to address limitations and clarify methods, it would be suitable for publication.

**Recommendation: **
The manuscript needs a major revision and re-peer review.

Reviewer #2: This article explores HCI researcher’s perceptions of open science practices through 18 semi-structured interviews with individuals skilled in qualitative and mixed methods. The authors state that five themes were identified: (1) knowledge of open science practices in the HCI community; (2) dynamics of peer review and open science practices; (3) potential benefits from open science; (4) barriers to adopt open science practices; and (5) incentives and recognition required to motivate. I agreed to review this article because I was excited by its focus and wanted to see whether common themes in past research were found to be similar, or contrasting, in the HCI field. Unfortunately, the paper left me feeling rather underwhelmed – the main issues are around the structure of the paper and its contents. At current, the paper does not feel that it is a finished, high-quality piece of writing – there are issues with the language, structure, and content. It also seems that it would be better placed in a HCI-specific journal rather than PLOS One which has a general readership. I now outline these in turn:

First, the Introduction is confusing with different sub-headings that do not differentiate from content that has gone before (e.g., “Related Work – Open Science Practices in HCI” follows on from a paragraph about OS practices in HCI”). It crucially excludes a lot of relevant literature about open science practices such as work that has explored barriers and challenges to O.S. through qualitative inquiry (e.g., Spitzer & Mueller (2023) and Sarafoglou et al. (2022) on barriers to preregistration).

Response: Thank you for your thoughtful suggestion. We have changed the structure of the Introduction to make it more clear for the readers. We have also worked on the related work section. We have divided the sections for better understanding and added all the references as suggested. Open Science Practices in HCI now has three sub sections. They are Preregistration and Study Design, Ethical Recruitment and Material Sharing, and Peer Review and Incentives.

Furthermore, the authors outline the various OS practices, such as preregistration, open materials, data and code, but miss out the practice of Registered Reports (see Chambers & Tzavella, 2022) – surely this practice is applicable to HCI researchers, too? (although uptake may be lower at current, see the work of Norris et al. and Silverstein et al. 2024). Many of the references at current are from HCI/CHI conferences etc. and I must admit that the paper reads as though it was submitted to a HCI/CHI conference with PLOS One as an afterthought – this is due to the lack of integration of more general open science, seminal, papers!

Response: Thank you for the suggestion. We have revised our related work section and have added a paragraph on “Preregistration and Study Design” to address this. We have added all the references as suggested.

Second, the Introduction needs to be better contextualise open science in the field of HCI – it appears from the themes that HCI researchers operate under different circumstances than other researchers. For example, they mention mainly presenting work at conferences, which will have different guidelines and incentives etc. than the pressures academics face in publishing their research or writing grants (I am not saying that these things don’t apply to HCI researchers, but it’s unclear from the Introduction what context and culture HCI researchers sit within). Furthermore, they mention developing prototypes which is unique to their field. Better contextualisation and discussion of these factors and potential barriers to O.S. would make for a lot stronger paper.

Response: Thank you for this constructive observation. We agree that better contextualizing the field of HCI and specifically, how its publishing culture, research practices, and methodological diversity shape engagement with open science is essential for grounding our study. We have added a section for this in the introduction.

Third, the outline of themes in the Results section does not then follow the same order when each theme is presented. Further, sub-themes are presented in one theme that overlap with another (e.g., under the theme “Knowledge of Open Research Practices by Early Career HCI researchers” there is a sub-theme named “Experiences with Peer Review from a Reviewer’s Standpoint”, but then there is another major theme named “Dynamics of Peer Review…”). Better integration and separation of the themes is therefore required.

Response: Thank you for this valuable observation. Our paper has five themes, they are "Knowledge of Open Research Practices by Early Career HCI Researchers ", "Dynamics of Peer Review and Open Science Practices in HCI Community ", "potential benefits from open science", "barriers to Adopt open science practices", and "Incentives and Recognition Required to Motivate". The sub-theme named “Experiences with Peer Review from a Reviewer’s Standpoint” is under “Dynamics of Peer Review and Open Science Practices in HCI Community”. We have added Figure 1 which represents the visualization of the themes for better clarity.

Dynamics of Peer Review and Open Science Practices in HCI Community is the major theme which has two sub themes. One is “Author Perspectives on Peer Review” and another one is “Experiences with Peer Review from a Reviewer’s Standpoint”. The first sub theme focuses on how participants experience peer review as authors—that is, how they perceive reviewer feedback, editorial decisions, and peer review culture when their own work is under evaluation. The second sub theme highlights how participants perform and understand the role of reviewer—including their sense of responsibility, interpretation of open science expectations, and decision-making as reviewers themselves. For better understanding we have changed the name of the 2nd sub theme. The current name is “Navigating Open Science as a Reviewer”. Figure 1 will add more clarity.

Finally, the Abstract and the last paragraph of the Introduction which outlines the results* paints an overall pessimistic overview of the themes and findings, focusing on the barriers mainly. However, many positive themes came out of this work which are not highlighted and explored. For example the Abstract states “Our findings highlight key barriers to the widespread adoption of data and materials sharing, and preregistration, namely: lack of clear incentives; cultural resistance…”, but what about the other themes of “knowledge of open science practices in the HCI community”, “dynamics of peer review and open science practices” and “potential benefits from open science” which paint a more optimistic picture of this field. The Abstract should be more balanced when discussing these themes, giving a broader overview. Furthermore, the overview of the findings should not just focus on these barriers, although they are important to circumvent.

Response: We acknowledge your feedback and we have highlighted the positive findings as well. We have changed the language of the abstract.

*The overview of the findings should be removed from the Introduction and included as the opening paragraph of the Discussion.

Response: Thank you for your feedback. We have included the paragraph as an opening section of Discussion.

Minor points:
The terms 'open science' and 'open research' are used interchangeably throughout the article.

Response: We have changed and made it uniform for the reader. Now it is represented as "open science" throughout.

Will a general authorship of PLOS One understand many of the abbreviations used throughout the article? For example, I recommend un-abbreviating HCI in the title (and also mentioning that this is U.S. researchers only), and un-abbreviating conferences so that a general readership knows what these are.

Response: Thank you for the suggestion. We have added all the full forms of all abbreviations used throughout the article. Changed the HCI into Human Computer Interaction in the Title with mentioning the study is for USA based candidates.

It would be beneficial to outline in the Abstract that the 18 researchers used mixed methods and qualitative methodology. There are different challenges/barriers to O.S. between quantitative and qualitative researchers. Discussion of this could also be included in the Introduction.

Response: We have added a section in the Related work section about the different challenges/barriers to O.S. between quantitative and qualitative researchers. Also mentioned in the abstract about 18 researchers used mixed methods and qualitative methodology as suggested.

The last sentence of the Abstract ("Future studies will include…") is confusing. Should it not be "should include"? What does "allowing more justifiable examples" mean?

Response: We have changed the language of the last sentence for clarity for the readers. The current statement is "Future studies will include HCI researchers with different levels of experience and from various countries, making the findings more well-rounded and representative".

The paper talks about how to incentivise open science through, e.g. better training. A new paper by Skubera et al. (2025) would help to make this point. See recommended references below.

Response: We have gone through all the recommended references and added them in proper location. Thank you for the suggestion.

The Introduction talks about ethics under open science, but this isn't an open science practice – in other words, all researchers have to adhere to ethical practice/clearance and this has existed way before the advent of open science practices. Perhaps you can rephrase this as improving equality, diversity, and accessibility within scientific fields, which O.S. aims to do. The work of FORRT may help here: https://forrt.org/equityinos/ and https://forrt.org/os-developing-world/ etc.

Response: We have changed the framing in the Introduction and added the reference suggested.

Structure – in the introduction you outline issues of replicability and reproducibility in the first paragraph, but also end this with showing increases in open data and open materials. This is confusing because these increases came about through the implementation of open science, but open science is mentioned in the next paragraph. It would be better to outline the issues of science and then the advent of open science and how this has led to positive improvements.

Response: We have changed the structure of the Introduction as suggested.

"Post-doctorate" should be Post Doctoral Research Assistant, or similar, in the first instance. It doesn't make sense as "Post-doctorate" alone.

Response: We have changed"Post-doctorate" to Post Doctoral Researcher as suggested.

Did you use the seminal work of Braun and Clarke for thematic analysis? How does the work of Blanford differ, what recommendations do they make that you implement?

Response: Yes, our study is grounded in the seminal work of Braun and Clarke on reflexive thematic analysis. We followed their six-phase framework, which includes, familiarization with the data, generating initial codes, searching for themes, reviewing themes, defining and naming themes, and producing the report. Their approach allowed us to systematically explore patterns in participants' interviews. In contrast, Blandford et al. provide more practical guidance specific to applied and systems-oriented research. Their work emphasizes the importance of aligning qualitative methods with design

goals, such as understanding user needs for system development. Blandford et al. are more focused on the rigor and relevance of qualitative findings for system design and evaluation.

One of the interpretations of themes suggests that participants suggest O.S. practices can reduce fraud (Page 10, Lines 380-385). However, the quote does not suggest this, and rather that assumption is made by the authors which goes beyond the data. I would revise this.

Response: Thank you for the suggestion. We have revised this section.

"Potential benefits from open science" – this theme is not relayed in the Abstract.

Response: We have added the benefits of open science in our Abstract.

The "Enhancing Training and Education" section of the Discussion would benefit from explicit resources that researchers can use. For example, there is no discussion of how journals are implementing the JARS-QUANT and JARS-QUAL guidelines: e.g. https://apastyle.apa.org/jars/quantitative. Again, this review of the positive changes that have been implemented through O.S. would be helpful:
https://royalsocietypublishing.org/doi/10.1098/rsos.241726

Response: Thank you for the suggestion. We have added this in the section called "The Role of Conferences and Journals" to address this.

Do you need to label the Discussion section as "Discussion" (rather than "Recommendation").

Response: We have changed the section as Discussion.

The Conclusion is very strong – this is written clearly and the same prose should be used throughout the article to ensure high-quality writing throughout.

Recommended references:
https://doi.org/10.1371/journal.pone.0281086
http://doi.org/10.1098/rsos.211997
https://doi.org/10.1111/bjop.12700
https://doi.org/10.31222/osf.io/w48yh
https://doi.org/10.1038/s41562-021-01193-7
https://doi.org/10.1098/rsos.241726

---

6. PLOS authors have the option to publish the peer review history of their article (what does this mean?). If published, this will include your full peer review and any attached files.

If you choose “no”, your identity will remain anonymous but your review may still be made public.

Do you want your identity to be public for this peer review? For information about this choice, including consent withdrawal, please see our Privacy Policy.

Reviewer #1: No

Reviewer #2: **Yes:** Dr Charlotte R. Pennington

[NOTE: If reviewer comments were submitted as an attachment file, they will be attached to this email and accessible via the submission site. Please log into your account, locate the manuscript record, and check for the action link "View Attachments". If this link does not appear, there are no attachment files.]

While revising your submission, please upload your figure files to the Preflight Analysis and Conversion Engine (PACE) digital diagnostic tool, https://pacev2.apexcovantage.com/. PACE helps ensure that figures meet PLOS requirements. To use PACE, you must first register as a user. Registration is free. Then, login and navigate to the UPLOAD tab, where you will find detailed instructions on how to use the tool. If you encounter any issues or have any questions when using PACE, please email PLOS at figures@plos.org. Please note that Supporting Information files do not need this step.
