# Supplementary material for: Open science practices among early-career human-computer interaction researchers in the US
Source: PLoS One. 2025 Nov 12;20(11):e0334692. doi: 10.1371/journal.pone.0334692 (PMC12611103; doi:10.1371/journal.pone.0334692)
Supplement: Attachment — Submitted filename: Comments.docx [file pone.0334692.s001.docx]

The manuscript provides a timely qualitative exploration of open science adoption barriers and perceptions within the HCI community. The study is well-motivated, methodologically sound, and offers actionable recommendations. However, some areas require clarification or improvement.

**Strengths:**

1. **Relevance**: Addresses a critical gap in understanding open science practices among early-career HCI researchers, aligning with broader reproducibility debates.

2. **Methodology**: Robust use of semi-structured interviews (n=18) and thematic analysis, with clear documentation (e.g., GitHub repository for protocols).

3. **Findings**: Identifies key barriers (e.g., lack of incentives, cultural resistance) and benefits (e.g., transparency, citations) with nuanced qualitative insights.

4. **Recommendations**: Practical suggestions (e.g., cultural shifts, conference mandates) are well-grounded in data.

**Weaknesses and Suggestions for Improvement:**

1. **Sample Limitations**:

- The study’s focus on U.S.-based researchers limits generalizability. Explicitly acknowledge this and recommend future cross-cultural studies.

- Consider adding demographic details (e.g., institutional types, subfields) to contextualize diversity within the sample.

2. **Self-Report Bias**:

- While noted in the abstract, emphasize how recall/social desirability biases might affect findings (e.g., overreporting openness). Suggest triangulation with behavioral data (e.g., artifact sharing rates).

3. **Thematic Analysis**:

- Clarify how inter-coder reliability was ensured during theme development. A brief description of the reconciliation process would strengthen rigor.

4. **Recommendations**:

- Expand on how to address intellectual property concerns (e.g., Creative Commons licensing examples).

- Discuss potential downsides of mandating practices (e.g., burden on qualitative researchers).

5. **Structural Notes**:

- The "Related Work" section could better integrate HCI-specific challenges (e.g., qualitative vs. quantitative transparency).

- Tables/visualizations (e.g., a summary of barriers/themes) would improve readability.

5. **Literature review**:

The manuscript should make greater use of other sources and studies that have addressed related topics. The following articles are among the relevant articles that need to be cited and used in this manuscript:

- Zarghani, Maryam; Nemati-Anaraki; Leila, Sedghi, Shahram; Noroozi Chakoli, Abdolreza; Rowhani-Farid, Anisa. (2023). "The Application of Open Science Potentials in Research Processes: A Comprehensive Literature Review". *Libri*. DOI: [10.1515/libri-2022-0007](https://doi.org/10.1515/libri-2022-0007)
- Zarghani, Maryam; Nemati-Anaraki, Leila; Sedghi, Shahram; Noroozi Chakoli, Abdolreza; Rowhani-Farid, Ansia. (2023). ”Iranian researchers’ perspective about concept and effect of open science on research publication”. *BMC Health Services Research*. 23, 437. DOI: 10.1186/s12913-023-09420-9

**Ethical and Data Considerations:**

- Ethics approval (IRB) and consent procedures are clearly documented.

- Data availability is commendable (GitHub), but ensure all links are functional at publication.

**Conclusion:**

This manuscript makes a valuable contribution to HCI and open science literature. With minor revisions to address limitations and clarify methods, it would be suitable for publication.

**Recommendation: **

The manuscript needs a major revision and re-peer review.
